# Effects of *Macleaya cordata* Extract on Blood Biochemical Indices and Intestinal Flora in Heat-Stressed Mice

**DOI:** 10.3390/ani12192589

**Published:** 2022-09-28

**Authors:** Mingcan Wang, Xiuqiong Huang, Yisong Liu, Jianguo Zeng

**Affiliations:** 1 Shanxi Key Laboratory for Modernization of TCVM, College of Veterinary Medicine, Shanxi Agricultural University, Taiyuan 030801, China; 2 Hunan Key Laboratory of Traditional Chinese Veterinary Medicine, Hunan Agricultural University, Changsha 410000, China

**Keywords:** heat stress, blood biochemical, gut microbiota, *Macleaya cordata* extract

## Abstract

**Simple Summary:**

Heat stress severely affects animals’ performance and welfare. *Macleaya cordata* extract is a potential anti-stress product. However, the effect of *Macleaya cordata* extract on heat stress in mice is unknown. This study discussed the effect of gavage *Macleaya cordata* extract on biochemical parameters and cecal flora in heat-stressed mice. This will provide a scientific basis for the application of *Macleaya cordata* extract in animal husbandry under heat stress conditions.

**Abstract:**

Heat stress (HS) leads to disturbance of homeostasis and gut microbiota. *Macleaya cordata* extract (MCE) has anti-inflammatory, antibacterial, and gut health maintenance properties. Still, the specific effects of MCE on blood biochemical indices and gut microbiota homeostasis in heat-stressed mice are not entirely understood. This study aimed to investigate the impact of MCE on blood biochemical indices and gut microbiota in heat-stressed mice. A control group (CON) (25 °C, n = 6) and HS group (42 °C, n = 6) were gavaged with normal saline 0.2 mL/g body weight/day, and HS plus MCE group (HS-MCE) (42 °C, n = 6) was gavaged with 5 mg MCE/kg/day. HS (2 h/d) on 8–14 d. The experiment lasted 14 days. The results showed that HS increased mice’ serum aspartate transaminase, alanine transferase activities, heat shock protein 70 level, and malondialdehyde concentrations, and decreased serum catalase and superoxide dismutase activities. HS also disrupted microbiota diversity and community structure in mice, increasing the Bacteroidetes and decreasing Firmicutes and *Lactobacillus*; however, MCE can alleviate the disturbance of biochemical indicators caused by HS and regulate the flora homeostasis. Furthermore, MCE was able to moderate HS-induced metabolic pathways changes in gut microbiota. The Spearman correlation analysis implied that changes in serum redox status potentially correlate with gut microbiota alterations in HS-treated mice.

## 1. Introduction

Heat stress (HS) is one of the everyday stressors [1]. HS reduces animal feed intake, affects nutrient absorption, nutrient utilization, and barrier function in the gut, increases the proliferation of pathogenic gut microbiota, and causes intestinal damage and translocation of pathogens and antigens into blood circulation, consequently seriously affecting animal growth performance and health [2]. Intestinal damage caused by HS is closely related to alterations in the gut microbiota [3]. In addition, HS results in the generation of reactive oxygen species (ROS) and an imbalance in the redox status [4,5], which in turn changes the intestine microbial community, leading to intestinal dysbiosis [6,7].

Animal gut microbiota participates in various physiological functions of the host, such as digestion, metabolism, immune regulation, energy conversion, mucosal development, and barrier maintenance [8,9,10,11]. The composition and function of the gut microbiota are critical to host health [12]. The gut microbiota can also influence the brain-gut axis to regulate host metabolic homeostasis, health, and behavior through microbiota-derived metabolites, hormones, and neurotransmitters [13]. The microbiota is predominantly present in specific intestinal segments, including the ileum and cecum. As the most diverse part of the gut, the cecum is colonized by unknown microorganisms, and the cecal microbiota is more stable than the ileal microbiota [14,15]. Typically, gut bacteria maintain relatively stable homeostasis. However, once this dynamic balance is disrupted, the intestinal flora’s quantity and proportion will be disturbed, resulting in dysbiosis and further pathological changes in the host [16].

Studies have shown that the bioactive compounds of many plants, including traditional Chinese medicine, improve intestinal flora eubiosis and ameliorate mucosal barrier dysfunction, especially during stressful conditions [2]. *Macleaya cordata* (family Papaveraceae) is a perennial herb and traditional Chinese medicine widely distributed in the south of China [17]. *Macleaya cordata* extract (MCE) is an orange-yellow powder made from the fruit pods of *Macleaya cordata* (Willd.) R. Br., which is a natural, pollution-free green product. The MCE standardized extraction process is referenced by Dong (2022) [18]. In our previous study, five species of MCE components were identified, including benzophenanthridine, benzyltetrahydroisoquinoline, protopine, protoberberine, and tetrahydroptotoberberine, and the specific components refer to Dong (2021) [19]. The main components of *Macleaya cordata* extract (MCE) are sanguinarine and chelerythrine [17]. Both sanguinarine and chelerythrine belong to benzophenanthridine. Benzophenanthridine alkaloids have the capacity to interfere with the transcription of DNA into RNA and therefore also affect protein synthesis, producing the bacteriostatic effect [20]. MCE has been reported to have a broad spectrum of biological activities, including antiviral, anti-inflammatory, antioxidative, detoxifying, and antimicrobial effects [17]. MCE is often used as a substitute for antibiotics in animal feed [21,22]. Sanguinarine and chelerythrine are considered excellent animal feed additives due to their unique pharmacological properties and health benefits [23,24]. Previous studies indicated that dietary supplementation with MCE could improve the growth performance of grass carp [25], weaned pigs [26], cattle [27], and broilers [21]. Dietary MCE increases intestinal *Lactobacillus* species and decreased Escherichia coli Shigella populations in weaned pigs [28]. MCE enhances the innate immune response in mice [29] and alleviates oxidative damage induced by weaning in the lower gut of young goats [30]. 

However, whether MCE attenuates HS-mediated dysregulation of homeostasis and gut microbiota in mice is unclear. Therefore, this study investigated the effects of MCE on blood biochemical markers and gut microbiota in heat-stressed mice.

## 2. Materials and Methods

### 2.1. Experimental Design and Diets

Four weeks old-male adult Kunming mice (18–22 g) were purchased from the Hunan Slake Jing da Experimental Animal Co., Ltd. (Changsha, China), with license number SCXK 2019-0016. Mice had free access to food and water. They were maintained on a 12 h light-dark cycle in conditions that controlled temperature (25 °C) and humidity (50 ± 5%). After an adaptation period of 1 week, 18 Kunming mice were randomly divided into three groups: control group (CON, 25 °C, n = 6), heat stress group (HS, 42 °C, n = 6), and heat stress plus MCE group (HS-MCE, n = 6). MCE was provided by Hunan Meikeda Biological Resources Co., Ltd. (Changsha, China). The CON group and HS group were intragastrically administered with normal saline 0.2 mL/g body weight/day, and the HS-MCE group was intragastrically administered 5 mg MCE/kg/day (purity 60%, CAS: 112025-60-2). The gavage was continued for a total of 14 days. The MCE references a previous study [31]. The HS and HS-MCE groups were subjected to HS (Temperature: 42 °C; Humidity: 70%) between 12:00 and 14:00 daily from the 8th to the 14th day, 2 hours a day. The test method was referred to Wang (2015) [32]. After the heat treatment, the animals were sacrificed immediately. Blood was drawn from the eye sockets of the mice. Serum and cecal content were collected and stored at −80 °C until use.

### 2.2. Serum ALT, AST Activities, and HSP70 Level

Serum levels of heat shock protein 70 (HSP70) and activities of aspartate aminotransferase (AST) and alanine transferase (ALT) were determined using commercial kits (Nanjing Jiancheng Bioengineering Institute, Nanjing, China) according to the manufacturer’s guidelines.

### 2.3. Serum Redox Status

Serum malondialdehyde (MDA) concentrations and glutathione peroxidase (GSH-Px), superoxide dismutase (SOD), peroxidase Catalase (CAT) activity were determined using commercial kits (Nanjing Jiancheng Bioengineering Institute, Nanjing, China) according to the manufacturer’s guidelines.

### 2.4. 16S rRNA Sequencing of Cecal Microbiota

Approximately 0.25 g of non-lyophilized cecal contents was weighed for DNA extraction. DNA was extracted from cecal contents using the SDS method, and DNA purity and concentration were determined by using NanoDrop2000 (V1.0, Thermo, Waltham, MA, USA). For sample amplification methods, see Liu (2020) [33]. MiSeq metagenomic sequencing was completed by Shanghai Meiji Biomedical Technology Co., Ltd. (Shanghai, China), and bacterial diversity index analysis was performed on its cloud platform.

### 2.5. Statistical Analysis

Data were analyzed using SPSS 24.0 software (V24.0, IBM Corp, Armonk, NY, USA). Statistical significance was determined by one-way analysis of variance (ANOVA) followed by Tukey’s multiple comparison test. Data are expressed as mean ± SEM. Values of *p* < 0.05 or *p* < 0.01 were applied to compare the statistical significance of differences.

## 3. Results

### 3.1. Effect of MCE on Serum HSP70, ALT, and AST Levels

In Table 1, heat exposure increased the HSP70, AST, and ALT levels in the serum of the HS group compared with the CON group (*p* < 0.05). MCE treatment reduced the serum HSP70, ALT, and AST levels in the HS-MCE group when compared with the HS group (*p* < 0.05).

### 3.2. Effect of MCE on Serum Redox Status

In Table 2, HS increased serum MDA concentrations and decreased SOD and CAT activity compared with the CON group (*p* < 0.05). MCE treatment decreased serum MDA concentrations and increased SOD and CAT activity compared with the HS group (*p* < 0.05). However, there were no remarkable changes in serum GSH-Px activity in the three groups (*p* > 0.05).

### 3.3. Effect of MCE on Bacterial OTU Number in the Cecal of Mice

**Figure 1:**
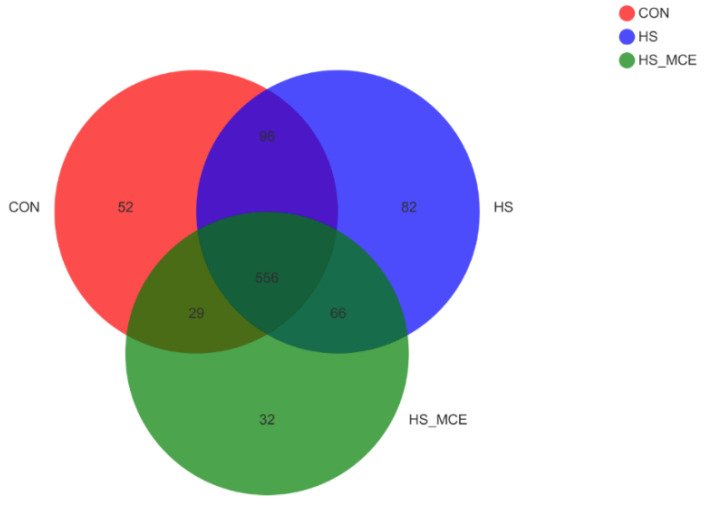
Effects of MCE on the number of cecal bacterial OTUs. The Venn diagram represents the number of shared and unique OTUs for the three groups, CON for the Control group, HS for the Heat Stress group, and HS-MCE for the Heat Stress plus *Macleaya cordata* extract group.

In Figure 1, Operational taxonomic units (OTUs) with 97% similarity cutoff [34,35] were clustered using UPARSE (version 7.1 http://drive5.com/uparse/, accessed on 10 May 2020) [34] and chimeric sequences were identified and removed. The taxonomy of each OTU representative sequence was analyzed by RDP Classifier version 2.2 [36] against the 16S rRNA database (Silva v138) using a confidence threshold of 0.7. There were 733, 800, and 683 OTUs found in the CON group, HS group, and HS-MCE group, respectively. Among them, 556 were identical. 

### 3.4. Effect of MCE on Bacterial Diversity in the Cecal of Mice

**Figure 2:**
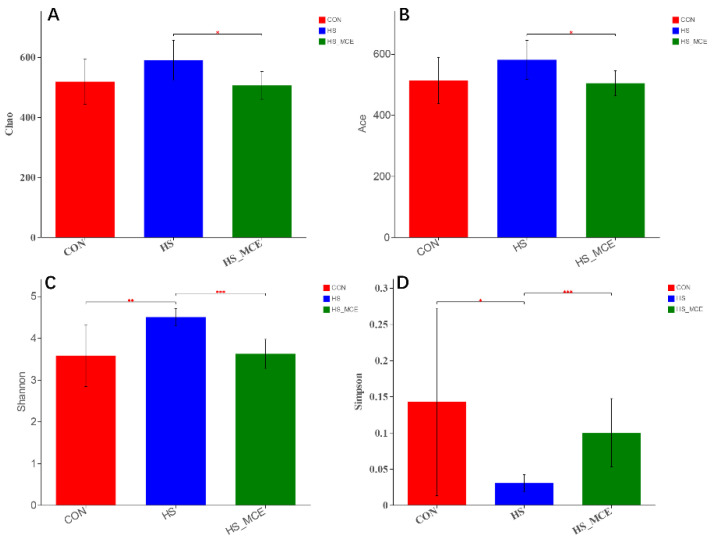
Effects of MCE on cecal bacterial Alpha diversity. (**A**) Chao index, (**B**) Ace index, (**C**) Shannon index, and (**D**) Simpson index. CON for the Control group, HS for the Heat Stress group, and HS-MCE for the Heat Stress plus *Macleaya cordata* extract group. * indicates statistically significant difference (*p* < 0.05). ** indicates extremely significant difference (*p* < 0.01). *** indicates very extremely significant difference (*p* < 0.001).

In Figure 2, Alpha diversity (Ace, Chao, Shannon, Simpson) analysis was performed using Mothur software (V 1.30.2, Michigan University, East Lansing, MI, USA). The results showed that HS significantly increased the Shannon index, but MCE treatment decreased the Ace, Chao, and Shannon indices in heat stressed mice compared with the CON group (*p* < 0.05). The Simpson index trend was opposite to the Shannon index. 

**Figure 3:**
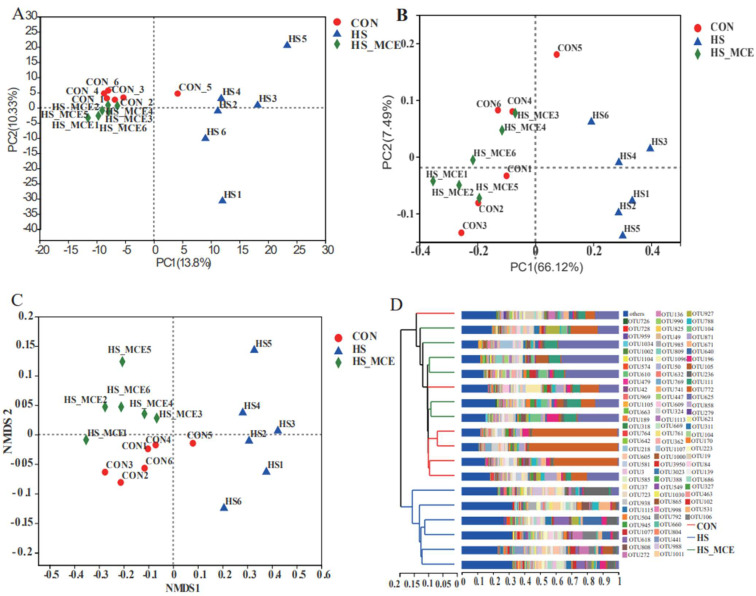
Effects of MCE on cecal bacterial Beta diversity. (**A**) PCA analysis, (**B**) PCoA analysis, (**C**) NMDS analysis, and (**D**) Hierarchical cluster analysis. CON for the Control group, HS for the Heat Stress group, and HS-MCE for the Heat Stress plus *Macleaya cordata* extract group.

Figure 3 shows the statistical analysis and graphing of microbial beta diversity (PCA, PCoA, NMDS, hierarchical clustering) using the R language (Version 3.3.1, Auckland University, Auckland City, New Zealand). According to the PCA analysis, there was clear clustering among the CON, HS, and HS-MCE groups. The HS group samples were relatively scattered compared with the CON and HS-MCE groups. The distance between the HS group and the other two groups is relatively large. In contrast, the distance between CON and HS-MCE groups is pretty close (Figure 3A). Moreover, we obtained a similar result in PCoA and NMDS (Figure 3B,C). A hierarchical cluster was used based on the UniFrac distance matrix to further investigate bacterial genes’ similarity and confirm the distinct microbial communities among the CON, HS, and HS-MCE groups. As shown in Figure 3D, CON group samples and HS-MCE group samples are clustered into one category, and HS samples are clustered into one category. 

### 3.5. Effect of MCE on the Community Composition of Gut Bacteria in Mice

**Figure 4:**
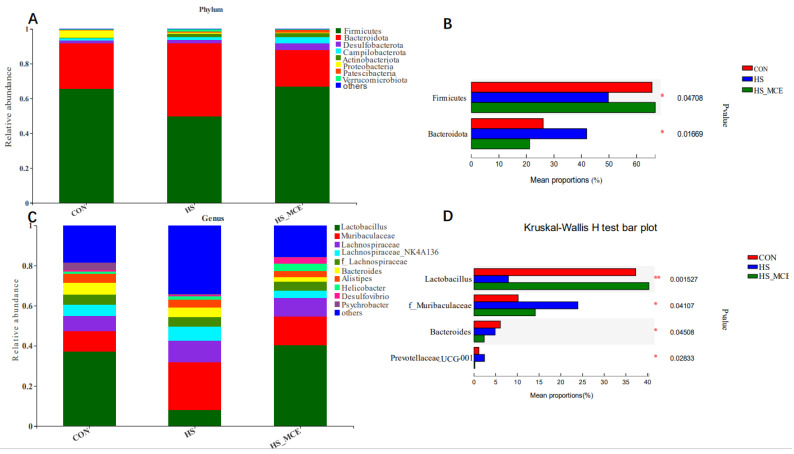
Effects of MCE on cecal bacterial abundance. Relative abundance of the cecal bacteria (**A**,**B**) at the phylum level and (**C**,**D**) at the genus level. CON for the Control group, HS for the Heat Stress group, and HS-MCE for the Heat Stress plus *Macleaya cordata* extract group. * indicates statistically significant difference (*p* < 0.05). ** indicates extremely significant difference (*p* < 0.01).

Figure 4 shows the statistical analysis and graphing of community composition (genus and phylum levels) using the R language (Version 3.3.1, Auckland University, Auckland City, New Zealand). Taxon abundance was consolidated at the genus and phylum levels for comparison across treatments. As shown in Figure 4A,B, the mice cecal microbial communities were dominated by Firmicutes, Bacteroidetes, Proteobacteria, and Actinobacteria. Firmicutes were the most abundant. At the phylum level, HS decreased Firmicutes abundance (*p* < 0.05), and increased Bacteroidetes abundance compared with the CON group (*p* < 0.05). As shown in Figure 4C,D, at the genus level, Lactobacillus abundance was significantly decreased, but Muribaculaceae, Prevotellaceae, Oscillospiraceae, and Alloprevotella abundance was markedly increased in the HS group (*p* < 0.05). However, at the phylum and genus levels, the changing trend of the cecal flora in the HS-MCE group was significantly alleviated compared with the HS group.

### 3.6. Changes in Gut Microbiota Function

**Figure 5:**
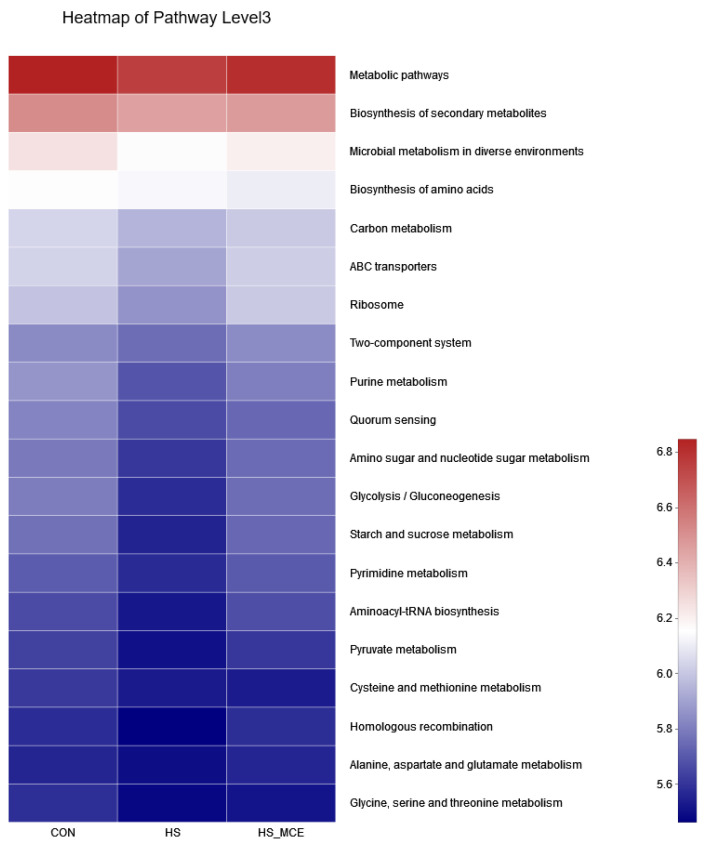
Predictive functional profiling to analyze the microbial communities among groups. CON for the Control group, HS for the Heat Stress group, and HS-MCE for the Heat Stress plus *Macleaya cordata* extract group.

In Figure 5, we used PICRUSt2 software (http://picrust.github.io/picrust/, accessed on 26 August 2022) to predict the metabolic pathways of the gut microbiota. At the third level of the KEGG hierarchy, the top 20 enrichment pathways are shown. Under the HS condition, the relative abundance of microbial metabolism in diverse environments, carbon metabolism, ABC transporters, ribosome, purine metabolism, quorum sensing, amino sugar and nucleotide sugar metabolism, glycolysis/gluconeogenesis, starch and sucrose metabolism, pyrimidine metabolism, aminoacyl-tRNA biosynthesis, and pyruvate metabolism decreased; after treatment with MCE, their relative abundance returned to normal levels.

### 3.7. Spearman Correlation Analysis

**Figure 6:**
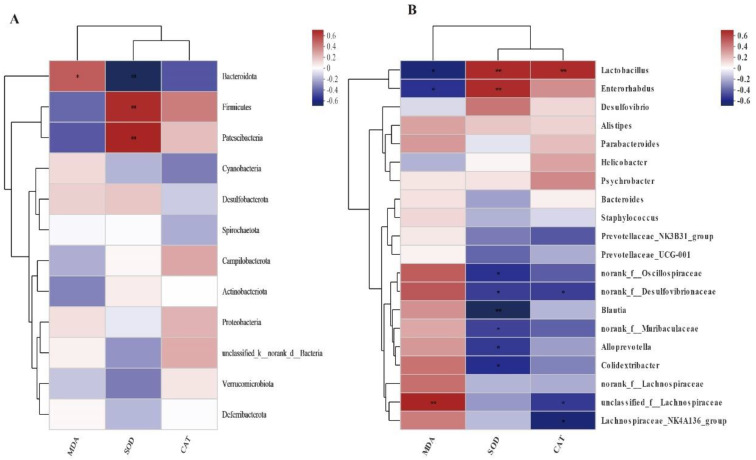
Spearman correlation analysis between serum MDA, SOD, CAT, and cecal flora genus level abundance. (**A**): phylum level (**B**): genus level. The Horizontal coordinate and Vertical coordinate are environmental factors and species, respectively. * *p* ≤ 0.05, ** *p* ≤ 0.01.

Figure 6 shows the statistical analysis and graphing of the Spearman correlation analysis using the R language (Version 3.3.1, Auckland University, Auckland City, New Zealand). The Spearman correlation analysis between the differential microbial species and antioxidant parameters was conducted to analyze the link between HS and intestinal microbiota. MDA was negatively associated with Bacteroidota, *Lactobacillus*, and *Enterorhabdus* (*p* < 0.05) and positively associated with *Lachnospiraceae* (*p* < 0.01). SOD activity was positively associated with the abundance of Firmicutes, Patescibacteria, *Lactobacillus*, and *Enterorhabdus* (*p* < 0.01), and negatively associated with Bacteroidota, *Blautia* (*p* < 0.01), and *Oscillospiraceae*, *Desulfovibrionaceae, Muribaculaceae*, *Alloprevotella*, *Colidextribacter* (*p* < 0.05). CAT activity was positively associated with the abundance of *Lactobacillus* (*p* < 0.01), and negatively associated with *Desulfovibrionaceae*, and *Lachnospiraceae* (*p* < 0.05).

## 4. Discussion

This experimental study shows that HS leads to abnormal blood biochemical indicators and intestinal flora disturbance in mice; while MCE can significantly reduce the serum HSP70, AST, ALT, and MDA concentrations and increase the serum SOD and CAT activities in mice induced by HS. In addition, MCE improved HS-induced microbiota homeostasis in mice, decreased microbiota alpha diversity (Ace, Chao, Shannon), improved microbiota structure, increased *Lactobacillus* abundance and decreased *Muribaculaceae*, *Prevotellaceae*, *Oscillospiraceae*, and *Alloprevotella* abundance at the genus level. MCE was able to moderate HS-induced metabolic pathways changes in the gut microbiota. The Spearman correlation analysis implied that changes in serum redox status potentially correlates with gut microbiota alterations in HS-treated mice.

The expression of heat stress proteins (HSPs) is closely associated with adapted thermotolerance against sudden heat shock [37]. HSPs, often referred to as chaperones, bind to unfolded or misfolded proteins and help restore their native conformation [38]. Studies have shown that HSPs are involved in cellular defense mechanisms that reduce heat-induced oxidative stress (OS) and inflammation [39]. HSP70 is the most conservative and typical family in the HSPs family and is widely expressed in tissues [40]. HSP70 overexpression is widely recognized as a hallmark of HS [41]. Our current research found that the serum HSP70 level was increased during HS exposure. In previous studies, HS led to a significant increase in the expression of HSP70 protein levels in testicular tissue [42] and liver tissue of mice [43]. Interestingly, the administration of MCE to heat-stressed mice reduced the HSP70 level, which is identical to the findings in sows and IUGR piglets [44]. Previous studies have shown that the expression level of HSP70 is significantly correlated with OS [45]. MCE played a significant role in improving antioxidant status through regulating the activity of serum SOD, GSH-Px and CAT [46]. MCE supplementation reduced MDA contents, increased SOD activities in both the ileum and jejunum, increased GSH-Px activity in the jejunum and attenuated oxidative damage in weaned goats [30]. Therefore, we thought that the effects of MCE on regulating Hsp70 levels of mice exposed to HS in this test may be due to the increase in SOD, and CAT activities. In addition, serum transaminases such as ALT and AST usually exist in the cytoplasm of hepatocytes and are commonly used as reliable indices to evaluate the damage degree to the liver [47]. HS causes liver damage, and transaminases are released from hepatocytes into the blood and transaminase activity increases in the serum when the liver is damaged. In this experiment, HS induced increased AST and ALT activities in the serum of mice, which is consistent with the previous study’s findings [48]. Research shows that MCE has a protective effect on piglet liver [49]. MCE (100 mg/L) can significantly inhibit the activities of ALT and AST in snail Oncomelania hupensis [50]. Combined metabolomic and network pharmacology approach analysis found that chelerythrine and sanguinarine in Corydalis saxicola Bunting are potentially active compounds in the treatment of liver fibrosis by modulating ALT activity [51]. As expected, MCE administration attenuated HS-mediated liver damage, evidenced by serum’s reduction in AST and ALT activities. The protective effect of MCE on the liver may be due to the inhibition of hepatic NAD(P)H quinone oxidoreductase activity [52] and the hepatic lipid peroxidation and inflammatory response [53] by chelerythrine. 

The body’s antioxidant system is in a state of homeostasis. The activities of serum SOD, GSH-Px, CAT and MDA are important indicators of the body’s antioxidant capacity [54]. The levels of SOD, GSH-Px and CAT reflect the ability of animals to scavenge ROS [55]. MDA is the final product of lipid peroxidation caused by ROS in animals, and its levels reflect the degree of damage caused by ROS to animals [56]. Too many ROS can disrupt the homeostasis of the antioxidant system and affect the health of the body. Studies have shown that HS causes excess ROS production, leading to OS [39]. In our current research, we also found that HS increased the mice’s serum MDA concentration, and decreased serum CAT and SOD activity compared with the CON group, which is consistent with the published literature [7,57]. It is interesting that MCE administration improved serum SOD and CAT activity and decreased MDA concentration compared with the HS group. MCE has strong antioxidant and ROS scavenging abilities, protecting cells from damage by inhibiting the production of ROS [49]. Adding MCE in maternal diets during late gestation can enhance the antioxidant level and alleviated OS of IUGR piglets while sows were exposed to HS [44]. MCE alleviates oxidative damage induced by weaning in the lower gut of young goats [30]. Previous research found that glucocorticoid receptor signaling represses the antioxidant response by inhibiting histone acetylation mediated by the transcriptional activator Nrf2 [58]. Sanguinarine and chelerythrine can induce the accumulation of glucocorticoid receptors in the nucleus with a concomitant reduction in cytosolic glucocorticoid receptors, and inhibit the binding of glucocorticoid to glucocorticoid receptors [59]. Therefore, MCE may enhance the antioxidant capacity of heat-stressed mice by inhibiting glucocorticoid receptor signaling and further inhibiting the transcriptional activator Nrf2-mediated histone acetylation. Indeed, sanguinarine can enhance the capacity of the enzymatic antioxidant defense system by activating the p38MAPK/Nrf2 pathway [60]. However, the mechanism by which MCE enhances antioxidant capacity in heat-stressed mice requires further study.

Richness and uniformity are two factors in species diversity analysis. The Chao, Ace, Simpson, and Shannon indices reflect the richness and diversity of the intestinal microbiota. In our current research, HS increased the Shannon index significantly compared with the CON group. In previous research, Patra found that HS significantly increased broilers ileal microbial alpha diversity, which was expressed as a higher number of observed species, Chao, and whole-tree phylogenetic diversity [4]. Wang confirmed that HS significantly increased observed species, whole-tree phylogenetic diversity, and Chao index in the ileum of broiler chickens [61]. However, it was also reported that HS reduced the cecal microbiome observed species, Chao, and Shannon index of heat-stressed broilers [62]. Moreover, HS did not alter alpha diversity, such as the Shannon index, of the gut microbiota in the cecum of laying hens [63]. The conflicting results of the intestinal microbiota alpha diversity might be associated with several factors, including the species, HS duration, exposure intensity, and gut segments. Interestingly, MCE treatment reduced the Chao, Shannon, and Ace index compared with the HS group. This is similar to previous findings that MCE treatment resulted in a significant decrease in ileal microbial diversity (Shannon index) in Xuefeng Black-boned Chicken [64]. In addition, the trend of OTUs number change is consistent with previous findings [65]. It suggests that MCE treatment increased the microbial diversity of cecum.

PCA was used to analyze the bacterial community structure. The sample distance distribution of the CON group and the HS-MCE group in the PCA diagram of this study is relatively concentrated. However, the sample distribution was relatively scattered in the HS group, and the sample distance was far from both the HS-MCE and CON groups, indicating that the bacterial community structure has a high similarity between the CON and HS-MCE groups, yet HS group samples are different. We obtained the same result in subsequent PCoA and NMDS analyses. The findings showed that HS destroyed the mice’s cecal bacterial structure, but MCE could reshape the cecal microbiota structure, which was consistent with previous studies [21,64,65].

Gut microbiota in mice was dominated by Firmicutes and Bacteroidetes [66]. Bacteroidetes are predominantly Gram-negative bacteria that help digest starch in food [67]. The ratio of Firmicutes and Bacteroidetes (F/B value) was commonly used to measure intestinal homeostasis [68]. Consistent with previous research findings [69]. In our current research, HS increased Bacteroidetes and decreased Firmicutes, resulting in abnormal F/B ratios, which suggests that HS leads to an imbalance of gut microbiota homeostasis. In addition, HS increased the abundance of *Muribaculaceae*, and *Prevotellaceae*, while decreasing the abundance of *Lactobacillus*. *Muribaculaceae* abundance has been correlated with increased short-chain fatty acids (SCFA) levels, which affect microglial density, morphology, and maturity [70]. *Prevotellaceae* has been demonstrated to be associated with obesity, and the *Prevotellaceae* are highly enriched in obese individuals [71]. *Lactobacillus* is recognized as a beneficial probiotic that inhibits pathogenic intestinal bacteria, promotes the development of T cells, enhances cellular immunity [72], and promotes the production of some essential vitamins and organic acids [73,74], indicating that *Lactobacillus* significantly contributes to improving immunity and nutrient absorption function. *Lactobacillus* is most affected by MCE, thus consistent with previous findings [67]. In this experiment, the content of *Lactobacillus* of MCE group was greater than others. MCE increased *Lactobacillus*, decreased *Muribaculaceae*, *Prevotellaceae*, and the F/B ratio returned to average, which was similar to previous studies’ results [21,75]. The inhibitory effects of different concentrations of MCE on the same flora were significantly different, and the effects of the same concentration of MCE on the flora of different species were also different. Studies have shown that MCE has the strongest inhibitory effect on Bacillus subtilis and Bacillus licheniformis, and the weakest inhibitory effect on *Lactobacillus* [76]. In this study, the reason why MCE did not inhibit the growth of *Lactobacillus* may be because the concentration of MCE did not reach the minimum inhibitory concentration of *Lactobacillus*. However, *Muribaculaceae* and *Prevotellaceae* were more sensitive to MCE and had an inhibitory effect. Moreover, the inhibitory effect of MCE on opportunistic pathogens such as *Muribaculaceae* and *Prevotellaceae* may indirectly lead to an increase in the predominant genus *Lactobacillus* due to the occupancy effect of the flora, and similar results can also be verified in previous studies [77]. The specific mechanism by which MCE leads to the growth of *Lactobacillus* requires further study.

**Figure 7:**
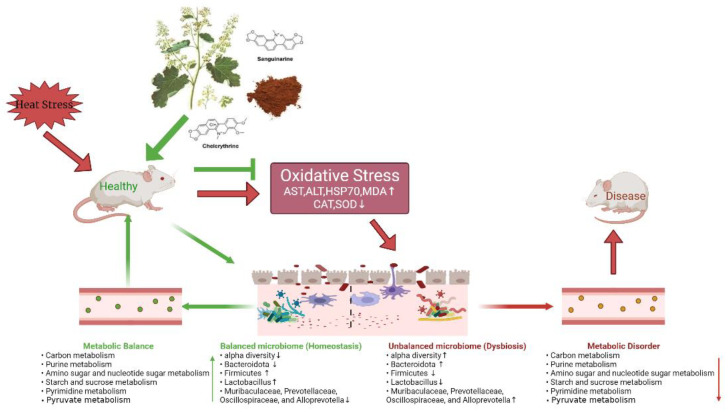
Possible mechanisms of MCE to relieve HS.

Previous studies have shown that MCE has anti-inflammatory and antioxidant properties [46,78]. We did detect a correlation between gut microbiota and antioxidant markers. SOD activity was positively associated with the Firmicutes, Patescibacteria, *Lactobacillus*, and *Enterorhabdus*. CAT activity was positively associated with the *Lactobacillus*. These data may support the hypothesis that MCE antioxidant properties are somewhat related to intestinal microbiota. In addition, KEGG predicted functional analysis of microbial communities revealed that the nutrient metabolism pathway (carbon metabolism, ABC transporters, ribosome, purine metabolism, amino sugar and nucleotide sugar metabolism, pyrimidine metabolism, and pyruvate metabolism) was suppressed by HS. Research shows that carbon metabolism is critical for persistence against OS [79]. ABC transporters play a central role in the defense against ethanol-induced oxidative damage in human neural cells [80]. Furthermore, ribosome [81], purine metabolism [82], sugar metabolism, amino acid metabolism, nucleotide metabolism [83], pyrimidine metabolism [84], and pyruvate metabolism [85] are also related to the body’s antioxidant capacity. MCE treatment significantly enhanced related metabolic pathways in heat-stressed mice. Therefore, we speculate that MCE may alleviate HS by promoting the growth and metabolism of bacterial groups such as Firmicutes, Patescibacteria, *Enterorhabdus* and *Lactobacillus*. MCE relieves HS in mice and the related mechanisms may involve the following factors shown in Figure 7.

## 5. Conclusions

In conclusion, the present study showed that MCE could alleviate HS-induced abnormal serum biochemical indices and intestinal flora disturbance in mice. Therefore, MCE could be a potential agent for preventing HS-related diseases. Of course, the underlying mechanism of MCE in relieving the OS induced by HS in a mouse model needs further investigation in the future.

## Figures and Tables

**Table 1:** Serum levels of HSP70, ALT, and AST.

Parameters	CON	Groups HS	HS-MCE
HSP70 (ng/mL)	7.97 ± 0.48 ^b^	14.24 ± 0.89 ^a^	10.63 ± 0.34 ^b^
AST (U/L)	43.88 ± 2.98 ^b^	69.48 ± 4.88 ^a^	47.39 ± 1.96 ^b^
ALT (U/L)	36.85 ± 1.96 ^b^	64.10 ± 3.82 ^a^	41.70 ± 2.47 ^b^

Note: HSP70, heat shock protein70; AST, aspartate aminotransferase; ALT, alanine aminotransferase; CON for the Control group, HS for the Heat Stress group, and HS-MCE for the Heat Stress plus *Macleaya cordata* extract group. Values are expressed as means ± SEM, n = 6. a,b Means within a row with different superscripts are different at *p* < 0.05.

**Table 2:** Serum of redox status.

Parameters	CON	Groups HS	HS-MCE
MDA (nmol/mL)	14.02 ± 0.90 ^b^	18.86 ± 1.19 ^a^	13.59 ± 1.11 ^b^
GSH-Px (mg/L)	577.50 ± 71.93	486.00 ± 36.08	585.50 ± 37.42
SOD (U/mL)	122.49 ± 4.68 ^a^	97.98 ± 4.03 ^b^	122.60 ± 2.81 ^a^
CAT (U/mL)	3.01 ± 0.21 ^a^	2.22 ± 0.06 ^b^	2.74 ± 0.08 ^a^

Note: MDA, malondialdehyde; GSH-Px, glutathione peroxidase; SOD, superoxide dismutase; CAT, catalase. CON for the Control group, HS for the Heat Stress group, and HS-MCE for the Heat Stress plus *Macleaya cordata* extract group. Values are expressed as means ± SEM, n = 6. a,b Means within a row with different superscripts are different at *p* < 0.05.

## Data Availability

The data presented in this study are available on request from the corresponding author.

## References

[1] Bouchama A., Knochel J.P. (2002). Heat Stroke. N. Engl. J. Med..

[2] Patra A.K., Kar I. (2021). Heat stress on microbiota composition, barrier integrity, and nutrient transport in gut, production performance, and its amelioration in farm animals. J. Anim. Sci. Technol..

[3] He J., He Y., Pan D., Cao J., Sun Y., Zeng X. (2019). Associations of Gut Microbiota With Heat Stress-Induced Changes of Growth, Fat Deposition, Intestinal Morphology, and Antioxidant Capacity in Ducks. Front. Microbiol..

[4] Zhang J.F., Hu Z.P., Lu C.H., Yang M.X., Zhang L.L., Wang T. (2015). Dietary curcumin supplementation protects against heat-stress-impaired growth performance of broilers possibly through a mitochondrial pathway1. J. Anim. Sci..

[5] Yang L., Tan G.-Y., Fu Y.-Q., Feng J.-H., Zhang M.-H. (2010). Effects of acute heat stress and subsequent stress removal on function of hepatic mitochondrial respiration, ROS production and lipid peroxidation in broiler chickens. Comp. Biochem. Physiol. C: Toxicol. Pharmacol..

[6] Liu X., Wu C., Han D., Liu J., Liu H., Jiang Z. (2019). Partially Hydrolyzed Guar Gum Attenuates d-Galactose-Induced Oxidative Stress and Restores Gut Microbiota in Rats. Int. J. Mol. Sci..

[7] Xia B., Wu W., Fang W., Wen X., Xie J., Zhang H. (2021). Heat stress-induced mucosal barrier dysfunction is potentially associated with gut microbiota dysbiosis in pigs. Anim. Nutr..

[8] Qin J., Li R., Raes J., Arumugam M., Burgdorf K.S., Manichanh C., Nielsen T., Pons N., Levenez F., Yamada T. (2010). A human gut microbial gene catalogue established by metagenomic sequencing. Nature.

[9] Gill S.R., Pop M., DeBoy R.T., Eckburg P.B., Turnbaugh P.J., Samuel B.S., Gordon J.I., Relman D.A., Fraser-Liggett C.M., Nelson K.E. (2006). Metagenomic Analysis of the Human Distal Gut Microbiome. Science.

[10] Sekirov I., Russell S.L., Antunes L.C.M., Finlay B.B. (2010). Gut Microbiota in Health and Disease. Physiol. Rev..

[11] Clemente J.C., Ursell L.K., Parfrey L.W., Knight R. (2012). The Impact of the Gut Microbiota on Human Health: An Integrative View. Cell.

[12] Bercik P., Collins S.M., Verdu E.F. (2012). Microbes and the gut-brain axis. Neurogastroenterol. Motil..

[13] Cao C., Chowdhury V.S., Cline M.A., Gilbert E.R. (2021). The Microbiota-Gut-Brain Axis during Heat Stress in Chickens: A Review. Front. Physiol..

[14] Borda-Molina D., Seifert J., Camarinha-Silva A. (2018). Current Perspectives of the Chicken Gastrointestinal Tract and Its Microbiome. Comput. Struct. Biotechnol. J..

[15] Kers J.G., Velkers F.C., Fischer E.A.J., Hermes G.D.A., Stegeman J.A., Smidt H. (2018). Host and Environmental Factors Affecting the Intestinal Microbiota in Chickens. Front. Microbiol..

[16] Thaiss C.A., Zmora N., Levy M., Elinav E. (2016). The microbiome and innate immunity. Nature.

[17] Lin L., Liu Y.-C., Huang J.-L., Liu X.-B., Qing Z.-X., Zeng J.-G., Liu Z.-Y. (2017). Medicinal plants of the genus *Macleaya* (*Macleaya cordata*, *Macleaya microcarpa*): A review of their phytochemistry, pharmacology, and toxicology. Phytother. Res..

[18] Dong Z., Tang S.-S., Ma X.-L., Li C.-H., Tang Z.-S., Yang Z.-H., Zeng J.-G. (2022). Preclinical safety evaluation of *Macleaya cordata* extract: A re-assessment of general toxicity and genotoxicity properties in rodents. Front. Pharmacol..

[19] Dong Z., Liu M., Zhong X., Ou X., Yun X., Wang M., Ren S., Qing Z., Zeng J. (2021). Identification of the Impurities in Bopu Powder^®^ and Sangrovit^®^ by LC-MS Combined with a Screening Method. Molecules.

[20] Cao P., Zhang Z.W., Leng D.J., Li X.Y., Li Y. (2016). Progress of antibacterial activity and antibacterial mechanism of isoquinoline alkaloids. China J. Chin. Mater. Medica.

[21] Liu Z.-Y., Wang X.-L., Ou S.-Q., Hou D.-X., He J.-H. (2020). Sanguinarine modulate gut microbiome and intestinal morphology to enhance growth performance in broilers. PLoS ONE.

[22] Chen J., Kang B., Zhao Y., Yao K., Fu C. (2018). Effects of natural dietary supplementation with *Macleaya cordata* extract containing sanguinarine on growth performance and gut health of early-weaned piglets. J. Anim. Physiol. Anim. Nutr..

[23] Li W., Li H., Yao H., Mu Q., Zhao G., Li Y., Hu H., Niu X. (2013). Pharmacokinetic and Anti-inflammatory Effects of Sanguinarine Solid Lipid Nanoparticles. Inflammation.

[24] Hu N.-X., Chen M., Liu Y.-S., Shi Q., Yang B., Zhang H.-C., Cheng P., Tang Q., Liu Z.-Y., Zeng J.-G. (2018). Pharmacokinetics of sanguinarine, chelerythrine, and their metabolites in broiler chickens following oral and intravenous administration. J. Vet. Pharmacol. Ther..

[25] Liu Y.-L., Zhong L., Chen T., Shi Y., Hu Y., Zeng J.-G., Liu H.-Y., Xu S.-D. (2020). Dietary sanguinarine supplementation on the growth performance, immunity and intestinal health of grass carp (Ctenopharyngodon idellus) fed cottonseed and rapeseed meal diets. Aquaculture.

[26] Li Y., Li H., Chu Q., Xu F., Liang T., Zhou B. (2018). *Macleaya cordata* extracts suppressed the increase of a part of antibiotic resistance genes in fecal microorganism of weaned pigs. Can. J. Anim. Sci..

[27] Michels A., Neumann M., Leão G.F.M., Reck A.M., Bertagnon H.G., Lopes L.S., De Souza A.M., Dos Santos L.C., Júnior E.S.S. (2018). Isoquinoline alkaloids supplementation on performance and carcass traits of feedlot bulls. Asian-Australasian J. Anim. Sci..

[28] Wang F., Yin Y., Yang M., Chen J., Fu C., Huang K. (2021). Effects of Combined Supplementation of *Macleaya cordata* Extract and Benzoic Acid on the Growth Performance, Immune Responses, Antioxidant Capacity, Intestinal Morphology, and Microbial Composition in Weaned Piglets. Front. Vet. Sci..

[29] Guan G., Ding S., Yin Y., Duraipandiyan V., Al-Dhabi N.A., Liu G. (2019). *Macleaya cordata* extract alleviated oxidative stress and altered innate immune response in mice challenged with enterotoxigenic Escherichia coli. Sci. China Life Sci..

[30] Chen K., Liu Y., Cheng Y., Yan Q., Zhou C., He Z., Zeng J., He J., Tan Z. (2020). Supplementation of Lactobacillus plantarum or *Macleaya cordata* Extract Alleviates Oxidative Damage Induced by Weaning in the Lower Gut of Young Goats. Animals.

[31] Li W., Li H., Mu Q., Zhang H., Yao H., Li J., Niu X. (2014). Protective effect of sanguinarine on LPS-induced endotoxic shock in mice and its effect on LPS-induced COX-2 expression and COX-2 associated PGE2 release from peritoneal macrophages. Int. Immunopharmacol..

[32] Wang F., Li Y., Cao Y., Li C. (2015). Zinc Might Prevent Heat-Induced Hepatic Injury by Activating the Nrf2-Antioxidant in Mice. Biol. Trace Element Res..

[33] Liu G., Zhu H., Ma T., Yan Z., Zhang Y., Geng Y., Zhu Y., Shi Y. (2020). Effect of chronic cyclic heat stress on the intestinal morphology, oxidative status and cecal bacterial communities in broilers. J. Therm. Biol..

[34] Edgar R.C. (2013). UPARSE: Highly accurate OTU sequences from microbial amplicon reads. Nat. Methods.

[35] Stackebrandt E., Goebel B.M. (1994). Taxonomic Note: A Place for DNA-DNA Reassociation and 16S rRNA Sequence Analysis in the Present Species Definition in Bacteriology. Int. J. Syst. Evol. Microbiol..

[36] Wang Q., Garrity G.M., Tiedje J.M., Cole J.R. (2007). Naïve Bayesian Classifier for Rapid Assignment of rRNA Sequences into the New Bacterial Taxonomy. Appl. Environ. Microbiol..

[37] Horowitz M. (2001). From molecular and cellular to integrative heat defense during exposure to chronic heat. Comp. Biochem. Physiol. Part A Mol. Integr. Physiol..

[38] Dahiya V., Buchner J. (2018). Functional principles and regulation of molecular chaperones. Adv. Protein Chem. Struct. Biol..

[39] Yun S.-H., Moon Y.-S., Sohn S.-H., Jang I.-S. (2012). Effects of Cyclic Heat Stress or Vitamin C Supplementation during Cyclic Heat Stress on HSP70, Inflammatory Cytokines, and the Antioxidant Defense System in Sprague Dawley Rats. Exp. Anim..

[40] Song Z., Cheng K., Zhang L., Wang T. (2017). Dietary supplementation of enzymatically treated Artemisia annua could alleviate the intestinal inflammatory response in heat-stressed broilers. J. Therm. Biol..

[41] Yu J., Bao E., Yan J., Lei L. (2008). Expression and localization of Hsps in the heart and blood vessel of heat-stressed broilers. Cell Stress Chaperon.

[42] Liu W., Leng J., Hou J., Jiang S., Wang Z., Liu Z., Gong X., Chen C., Wang Y. (2020). Saponins derived from the stems and leaves of *Panax ginseng* attenuate scrotal heat-induced spermatogenic damage via inhibiting the MAPK mediated oxidative stress and apoptosis in mice. Phytotherapy Res..

[43] Li S.-Q., Li R.-F., Xi S.-M., Hu S., Jia Z.-Q., Li S.-P., Wen X.-L., Song Y.-K., Li S., Li S.-P. (2011). Systematical analysis of impacts of heat stress on the proliferation, apoptosis and metabolism of mouse hepatocyte. J. Physiol. Sci..

[44] Li Y., Fan M., Qiu Q., Wang Y., Shen X., Zhao K. (2022). Nano-selenium and *Macleaya cordata* Extracts Improved Immune Function and Reduced Oxidative Damage of Sows and IUGR Piglets After Heat Stress of Sows in Late Gestation. Biol. Trace Element Res..

[45] Kurashova N.A., Madaeva I.M., Kolesnikova L. (2019). Expression of heat shock proteins HSP70 under oxidative stress. Adv. Gerontol..

[46] Li Y., He J., Zhang Q., Li L., Wang Y. (2021). Nano-Molybdenum and *Macleaya cordata* Extracts Improved Antioxidant Capacity of Grazing Nanjiang Brown Goats on Copper and Cadmium-Contaminated Prairies. Biol. Trace Element Res..

[47] Attia Y.A., El-Hamid A.E.A., Abdallah A.A., Berikaa M.A., El-Gandy M.F., Sahin K., Abou-Shehema B.M. (2018). Effect of betaine, vitamin C and vitamin E on egg quality, hatchability, and markers of liver and renal functions in dual-purpose breeding hens exposed to chronic heat stress. Europ. Poult. Sci.

[48] Chen Y., Jiang W., Liu X., Du Y., Liu L., Ordovas J.M., Lai C.-Q., Shen L. (2020). Curcumin supplementation improves heat-stress-induced cardiac injury of mice: Physiological and molecular mechanisms. J. Nutr. Biochem..

[49] Liu C., Li Y., Li H., Wang Y., Zhao K. (2021). Nano-Selenium and *Macleaya cordata* Extracts Improved Immune Functions of Intrauterine Growth Retardation Piglets under Maternal Oxidation Stress. Biol. Trace Element Res..

[50] Ke W., Lin X., Yu Z., Sun Q., Zhang Q. (2017). Molluscicidal activity and physiological toxicity of *Macleaya cordata* alkaloids components on snail Oncomelania hupensis. Pestic. Biochem. Physiol..

[51] Liu X.-W., Tang C.-L., Zheng H., Wu J.-X., Wu F., Mo Y.-Y., Liu X., Zhu H.-J., Yin C.-L., Cheng B. (2018). Investigation of the hepatoprotective effect of Corydalis saxicola Bunting on carbon tetrachloride-induced liver fibrosis in rats by 1H-NMR-based metabonomics and network pharmacology approaches. J. Pharm. Biomed. Anal..

[52] Huang C.-Y., Huang Y.-J., Zhang Z.-Y., Liu Y.-S., Liu Z.-Y. (2021). Metabolism and Tissue Distribution of Chelerythrine and Effects of *Macleaya cordata* Extracts on Liver NAD(P)H Quinone Oxidoreductase. Front. Vet. Sci..

[53] Zeng J., Xiao L., Wang Y., Liu L., Zhong M., He X., Liu Y. (2012). Experimental study on antagonizing liver fibrosis of *Macleaya cordata* extract. Chin. J. Exp. Tradit. Med. Formulae.

[54] Li Y., Liu H., He J., Shen X., Zhao K., Wang Y. (2022). The Effects of Oral Administration of Molybdenum Fertilizers on Immune Function of Nanjiang Brown Goat Grazing on Natural Pastures Contaminated by Mixed Heavy Metal. Biol. Trace Element Res..

[55] Han Q., Zhang J., Sun Q., Xu Y., Teng X. (2020). Oxidative stress and mitochondrial dysfunction involved in ammonia-induced nephrocyte necroptosis in chickens. Ecotoxicol. Environ. Saf..

[56] Li Y., He J., Luo L., Wang Y. (2022). The Combinations of Sulfur and Molybdenum Fertilization Improved Antioxidant Capacity in Grazing Nanjiang Brown Goat. Biol. Trace Element Res..

[57] Wen C., Leng Z., Chen Y., Ding L., Wang T., Zhou Y. (2021). Betaine Alleviates Heat Stress-Induced Hepatic and Mitochondrial Oxidative Damage in Broilers. J. Poult. Sci..

[58] Alam M., Okazaki K., Nguyen L.T.T., Ota N., Kitamura H., Murakami S., Shima H., Igarashi K., Sekine H., Motohashi H. (2017). Glucocorticoid receptor signaling represses the antioxidant response by inhibiting histone acetylation mediated by the transcriptional activator NRF2. J. Biol. Chem..

[59] Dvořák Z., Vrzal R., Maurel P., Ulrichová J. (2006). Differential effects of selected natural compounds with anti-inflammatory activity on the glucocorticoid receptor and NF-κB in HeLa cells. Chem. Interact..

[60] Vrba J., Orolinova E., Ulrichova J. (2012). Induction of heme oxygenase-1 by *Macleaya cordata* extract and its constituent sanguinarine in RAW264.7 cells. Fitoterapia.

[61] Wang X., Feng J., Zhang M., Li X., Ma D., Chang S. (2018). Effects of high ambient temperature on the community structure and composition of ileal microbiome of broilers. Poult. Sci..

[62] Sohail M.U., Hume M.E., Byrd J.A., Nisbet D.J., Shabbir M.Z., Ijaz A., Rehman H. (2015). Molecular analysis of the caecal and tracheal microbiome of heat-stressed broilers supplemented with prebiotic and probiotic. Avian Pathol..

[63] Xing S., Wang X., Diao H., Zhang M., Zhou Y., Feng J. (2019). Changes in the cecal microbiota of laying hens during heat stress is mainly associated with reduced feed intake. Poult. Sci..

[64] Guo S., Liu L., Lei J., Qu X., He C., Tang S., Xiao B., Li P., Gao Q., Lan F. (2021). Modulation of intestinal morphology and microbiota by dietary *Macleaya cordata* extract supplementation in Xuefeng Black-boned Chicken. Animal.

[65] Li X., Zhang C., Hui H., Tan Z. (2021). Effect of Gegenqinlian decoction on intestinal mucosal flora in mice with diarrhea induced by high temperature and humidity treatment. 3 Biotech.

[66] Schneeberger P.H.H., Coulibaly J.T., Panic G., Daubenberger C., Gueuning M., Frey J.E., Keiser J. (2018). Investigations on the interplays between Schistosoma mansoni, praziquantel and the gut microbiome. Parasites Vectors.

[67] Stevenson D.M., Weimer P.J. (2007). Dominance of Prevotella and low abundance of classical ruminal bacterial species in the bovine rumen revealed by relative quantification real-time PCR. Appl. Microbiol. Biotechnol..

[68] Shin N.-R., Whon T.W., Bae J.-W. (2015). Proteobacteria: Microbial signature of dysbiosis in gut microbiota. Trends Biotechnol..

[69] Zhu L., Liao R., Wu N., Zhu G., Yang C. (2018). Heat stress mediates changes in fecal microbiome and functional pathways of laying hens. Appl. Microbiol. Biotechnol..

[70] Erny D., Hrabě de Angelis A.L., Jaitin D., Wieghofer P., Staszewski O., David E., Keren-Shaul H., Mahlakoiv T., Jakobshagen K., Buch T. (2015). Host microbiota constantly control maturation and function of microglia in the CNS. Nat. Neurosci..

[71] Zhang H., DiBaise J.K., Zuccolo A., Kudrna D., Braidotti M., Yu Y., Parameswaran P., Crowell M.D., Wing R., Rittmann B.E. (2009). Human Gut Microbiota in Obesity and after Gastric Bypass. Proc. Natl. Acad. Sci. USA.

[72] Zhao X.P., Xiao X.Y., Cai R., Tan Z.J., Li D.D. (2014). The progress in research of constipation-related gut microbes. Chin. J. Microecol..

[73] Neal-McKinney J.M., Lu X., Duong T., Larson C.L., Call D.R., Shah D.H., Konkel M.E. (2012). Production of Organic Acids by Probiotic Lactobacilli Can Be Used to Reduce Pathogen Load in Poultry. PLoS ONE.

[74] LeBlanc J.G., Milani C., de Giori G.S., Sesma F., van Sinderen D., Ventura M. (2012). Bacteria as vitamin suppliers to their host: A gut microbiota perspective. Curr. Opin. Biotechnol..

[75] Lee K.-W., Kim J.-S., Oh S.-T., Kang C.-W., An B.-K. (2015). Effects of Dietary Sanguinarine on Growth Performance, Relative Organ Weight, Cecal Microflora, Serum Cholesterol Level and Meat Quality in Broiler Chickens. J. Poult. Sci..

[76] Shaohang Z.P.W., Qijun W., Huayun L., Sijing J., Guimin Z. (2018). Effects of veterinary boluohui powder on the growth of commonly used probiotics. Feed. Ind..

[77] Huang P., Zhang Y., Xiao K., Jiang F., Wang H., Tang D., Liu D., Liu B., Liu Y., He X. (2018). The Chicken Gut Metagenome and the Modulatory Effects of Plant-Derived Benzylisoquinoline Alkaloids. Microbiome.

[78] Dong Z., Tang S.-S., Li C.-H., Tang Z.-S., Yang Z.-H., Zeng J.-G. (2022). Safety assessment of MPTA: An oral acute and 90-day sub-chronic toxicity study in Sprague-Dawley rats. Regul. Toxicol. Pharmacol..

[79] Shimizu K., Matsuoka Y. (2019). Redox rebalance against genetic perturbations and modulation of central carbon metabolism by the oxidative stress regulation. Biotechnol. Adv..

[80] Vishwakarma S.K., Fatima N., Lakkireddy C., Raju N., Bardia A., Sandhya A., Paspala S.A.B., Satti V., Khan A.A. (2018). Role of drug transporters and heat shock proteins during ethanol exposure to human neural precursor cells and its lineages. Tissue Cell.

[81] Fisunov G.Y., Evsyutina D.V., Garanina I.A., Arzamasov A.A., Butenko I.O., Altukhov I.A., Nikitina A.S., Govorun V.M. (2017). Ribosome profiling reveals an adaptation strategy of reduced bacterium to acute stress. Biochimie.

[82] Glantzounis G.K., Tsimoyiannis E.C., Kappas A.M., Galaris D.A. (2005). Uric Acid and Oxidative Stress. Curr. Pharm. Des..

[83] Zhang Z., Liang Z.C., Zhang J.H., Tian S.L., Le Qu J., Tang J.N., De Liu S. (2018). Nano-sized TiO_2_ (nTiO_2_) induces metabolic perturbations in Physarum polycephalum macroplasmodium to counter oxidative stress under dark conditions. Ecotoxicol. Environ. Saf..

[84] Gimadieva A.R., Myshkin V.A., Mustafin A.G., Chernyschenko Y.N., Fattakhov A.K., Abdrakhmanov I.B., Tolstikov G.A. (2013). 5-amino-6-methyluracil is a promising pyrimidine antioxidant. Dokl. Biol. Sci..

[85] Cappel D.A., Deja S., Duarte J.A., Kucejova B., Iñigo M., Fletcher J.A., Fu X., Berglund E.D., Liu T., Elmquist J.K. (2019). Pyruvate-Carboxylase-Mediated Anaplerosis Promotes Antioxidant Capacity by Sustaining TCA Cycle and Redox Metabolism in Liver. Cell Metab..

